# Interactions between amiodarone and the hERG potassium channel pore determined with mutagenesis and *in silico* docking

**DOI:** 10.1016/j.bcp.2016.05.013

**Published:** 2016-08-01

**Authors:** Yihong Zhang, Charlotte K. Colenso, Aziza El Harchi, Hongwei Cheng, Harry J. Witchel, Chris E. Dempsey, Jules C. Hancox

**Affiliations:** a School of Physiology and Pharmacology and Cardiovascular Research Laboratories, Medical Sciences Building, University of Bristol, University Walk, Bristol BS8 1TD, UK; b School of Biochemistry, Medical Sciences Building, University of Bristol, University Walk, Bristol BS8 1TD, UK; c Brighton and Sussex Medical School, University of Sussex, Falmer BN1 9PX, UK

**Keywords:** AF, atrial fibrillation, diLQTS, drug induced long QT syndrome, DEA, desethylamiodarone, HEK, human embryonic kidney, hERG, *human-Ether-à-go-go-Related Gen*e, IC_50_, half-maximal inhibitory concentration, *I*_hERG_, ionic current carried by hERG potassium channels, *I*_Kr_, rapid delayed rectifier potassium current, *I*_Ks_, slow delayed rectifier potassium current, LQTS, long QT syndrome, Amiodarone, Antiarrhythmic, hERG, *I*_Kr_, Long QT, QT interval

## Abstract

The antiarrhythmic drug amiodarone delays cardiac repolarisation through inhibition of *hERG*-encoded potassium channels responsible for the rapid delayed rectifier potassium current (*I*_Kr_). This study aimed to elucidate molecular determinants of amiodarone binding to the hERG channel. Whole-cell patch-clamp recordings were made at 37 °C of ionic current (*I*_hERG_) carried by wild-type (WT) or mutant hERG channels expressed in HEK293 cells. Alanine mutagenesis and ligand docking were used to investigate the roles of pore cavity amino-acid residues in amiodarone binding. Amiodarone inhibited WT outward *I*_hERG_ tails with a half-maximal inhibitory concentration (IC_50_) of ∼45 nM, whilst inward *I*_hERG_ tails in a high K^+^ external solution ([K^+^]_e_) of 94 mM were blocked with an IC_50_ of 117.8 nM. Amiodarone’s inhibitory action was contingent upon channel gating. Alanine-mutagenesis identified multiple residues directly or indirectly involved in amiodarone binding. The IC_50_ for the S6 aromatic Y652A mutation was increased to ∼20-fold that of WT *I*_hERG_, similar to the pore helical mutant S624A (∼22-fold WT control). The IC_50_ for F656A mutant *I*_hERG_ was ∼17-fold its corresponding WT control. Computational docking using a MthK-based hERG model differentiated residues likely to interact directly with drug and those whose Ala mutation may affect drug block allosterically. The requirements for amiodarone block of aromatic residues F656 and Y652 within the hERG pore cavity are smaller than for other high affinity *I*_hERG_ inhibitors, with relative importance to amiodarone binding of the residues investigated being S624A ∼ Y652A > F656A > V659A > G648A > T623A.

## Graphical abstract

**Figure:**
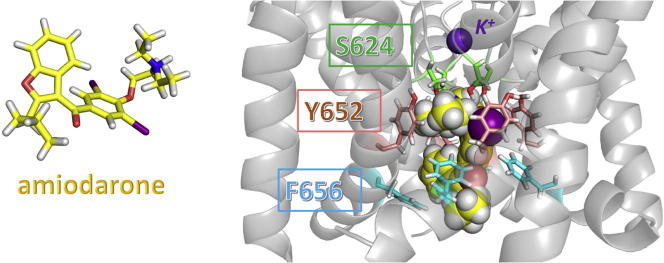

## 1 Introduction

The benzofuran-based Class III antiarrhythmic drug amiodarone is used in the treatment of both supraventricular and ventricular arrhythmias [1], [2]. It is recommended for the pharmacological cardioversion of recent onset atrial fibrillation (AF) in patients with structural heart disease, may enhance the effectiveness of direct current cardioversion of AF and can be useful for long-term rate-control in patients with a history of AF [3]. Intravenous amiodarone is the most effective pharmacological approach for managing life-threatening ventricular arrhythmias and is valuable in the treatment of cardiac arrest [4], [5]. Amiodarone’s comparatively favourable safety profile is likely to result from the fact that the drug has multiple cardiac ion channel-blocking effects (on K^+^_,_ Na^+^ and Ca^2+^ channels) as well as β-adrenoceptor blocking activity (for reviews see [1], [2]).

Rapid and slow delayed rectifier K^+^ currents (*I*_Kr_ and *I*_Ks_ respectively) are important contributors to cardiac action potential repolarisation [6], [7]. Short term administration of amiodarone preferentially inhibits ventricular *I*_Kr_ over *I*_Ks_, a result replicated in experiments on recombinant “hERG” and “KCNQ1 + KCNE1” channels [8], for nomenclature see [9]. Amiodarone was first demonstrated to inhibit hERG (*human-Ether-à-go-go-Related Gene*) encoded channels in 1999 [10]. hERG current (*I*_hERG_) measurements from *Xenopus* oocytes showed a half-maximal inhibitory concentration (IC_50_) of 9.8 μM, with suggested mixed channel-state (closed, open, inactivated channel) block [10]. Similar to other drugs, amiodarone’s *I*_hERG_ blocking potency is greater when the drug is tested on mammalian cell lines expressing hERG [11], [12], [13], [14], [15]. With mammalian expression systems, *I*_hERG_ IC_50_ values for amiodarone of between ∼26 and 300 nM were reported [12], [13], [14], [16], [17] and its metabolite desethyl-amiodarone (DEA) has been shown also to inhibit *I*_hERG_, with an IC_50_ of ∼160 nM [14]. It is likely, therefore, that *I*_hERG_/*I*_Kr_ blockade contributes to the acute clinical effects of amiodarone administration and that an inhibitory action of DEA additionally contributes to the chronic actions of the drug [14].

hERG channels are of particular pharmacological interest as they have a high susceptibility to pharmacological blockade by diverse cardiac and non-cardiac drugs, an action that is strongly associated with drug-induced Long QT Syndrome (diLQTS) [18], [19]. The channel’s ability to interact with diverse drugs is attributed to structural features of the channel that include a comparatively large inner cavity and the presence of aromatic amino-acid residues (Y652 and F656) in the S6 domain that favour drug interactions [18], [19]. For example, alanine mutants of Y652 and F656 have been shown to increase the IC_50_ for hERG block by the methansulphonanilide MK-499 by 94-fold and 650-fold respectively [20], and they also have a profound effect on the inhibitory actions effects of the related drugs dofetilide and E-4031 [21]. For many (typically high affinity) drugs hERG channel inactivation also appears to contribute to the drug-channel interaction [13], [18], [19], [22].

Amiodarone appears to differ from canonical *I*_hERG_ inhibitors in the extent to which channel inactivation influences blocking potency [13]. In a direct comparison with E-4031, amiodarone’s action was impaired less than that of E-4031 by attenuated-inactivation mutants [13]. Moreover, the effects of a profound blocking concentration of amiodarone (10× IC_50_ for wild-type hERG) have been reported to be only partially attenuated by mutation at Y652, whilst a concentration blocking WT *I*_hERG_ by ∼90% has been suggested to be relatively little affected by mutation at F656 [12]. These observations raise the possibility that binding determinants of amiodarone inhibition of hERG channels may be qualitatively or quantitatively different from those for canonical high affinity hERG inhibitors. The present study was undertaken to elucidate the nature of the interaction between amiodarone and the hERG channel, through mutagenesis of amino acids from the S6 and pore-helix regions that line the channel’s inner cavity together with *in silico* docking and molecular dynamics simulations. The results obtained show that, whilst in common with other drugs amiodarone binds within the hERG channel inner cavity, the roles of S6 aromatic residues are quantitatively smaller than for high affinity selective *I*_Kr_/*I*_hERG_ inhibitors [20], [21] and that other residues contribute significantly to amiodarone’s blocking action.

## 2 Materials and methods

### 2.1 Mutagenesis

An alanine-scanning approach was used to examine most of the individual residues from the S6 helix and the H5 pore/selectivity filter for possible interaction with amiodarone. The residues examined with the alanine scan are highlighted in Fig. 3A. Alanine was chosen because of its small size and its likely ability to minimise interruptions in secondary structure in tightly packed regions of the channel and this approach is an established one for studying structural determinants of hERG channel blockade [20], [21], [23]. Alanine mutants of hERG at the base of the pore helices near the selectivity filter (T623A, S624A, V625A) and the S6 helix (L646A, I647A, G648A, S649A, M651A, S654A, G657A, N658A, V659A, S660A, I663A, Q664A, R665A, L666A and Y667A) were constructed using the QuickChange® site-directed mutagenesis kit (Stratagene, La Jolla, CA) as previously reported ([24], [25], [26], see Table 1 for primers used). L622A, L650A, I655A, and I662A are excluded from this list as they do not express channels that conduct currents [20], [23]. A pair of complementary oligonucleotide primers (forward primers and reverse primers were synthesised by Sigma-Genosys, Haverhill, UK, see Table 1) were used in a PCR (95 °C for 1 min, 60 °C for 1 min, 68 °C for 16 min for 18 cycles) using hERG in a modified pcDNA3.0 vector as a DNA template. A DpnI (New England Biolabs Ltd, Herts, UK) digest of the PCR mix was then performed for 1 h at 37 °C. Competent DH5α Escherichia coli (Invitrogen, Paisley, UK) were transformed using standard procedures. Mutations were confirmed by sequencing the entire open reading frame (Eurofins MWG Operon, Ebersberg, Germany).

### 2.2 Maintenance of mammalian cell lines and cell transfection

Experiments on wild-type hERG were performed on a cell line (Human Embryonic Kidney; HEK 293) stably expressing hERG (generously donated by Dr. Craig January, University of Wisconsin). HEK 293 cell lines stably expressing mutant F656A and Y652A hERG were created in our laboratory using standard techniques: appropriately mutated hERG sequences were subcloned into a hERG expression vector (based on pIRES1hyg) into the BstEII/Sse8387I sites of hERG; the expression constructs were transfected using Fugene (Roche Diagnostics, West Sussex, UK) into HEK 293 cells, selected, subcloned, and assayed for hERG expression by immunofluorescence (using Alomone APC-016, Jerusalem, Israel) followed by electrophysiological validation [27]. Cells were passaged using enzyme free cell dissociation solution (Millipore, Watford, UK) and plated onto sterilised 13-mm glass coverslips in 40-mm petri dishes containing a modification of Dulbecco minimum essential medium with Glutamax-1 (DMEM; Invitrogen, Paisley, UK). This was supplemented with 10% fetal bovine serum, 50 μg/mL gentamycin (Invitrogen, Paisley, UK), and 400 μg/mL geneticin (G418, Invitrogen, Paisley, UK) for WT or 100 μg/mL of hygromycin for Y652A and F656A [14], [24], [25], [26], [27]. For other mutants, HEK293 cells (ECACC, Porton Down, UK) were transiently transfected with cDNA plasmids using Lipofectamine 2000 (Invitrogen, Paisley, UK) according to the manufacturer’s instructions. Expression plasmid encoding CD8 was also added (in pIRES, donated by Dr. I Baró, University of Nantes, France) as a marker for successful transfection. Recordings were performed 12–72 h after transfection. Successfully transfected cells (positive to CD8) were identified using Dynabeads® (Invitrogen, Paisley, UK) [24], [25], [26].

### 2.3 Solutions, electrophysiological recordings, experimental protocol and data analysis

Once in the recording chamber, cells were superfused with normal Tyrode’s solution containing (in mM): 140 NaCl, 4 KCl, 2.5 CaCl_2_, 1 MgCl_2_, 10 Glucose, and 5 HEPES (titrated to pH of 7.45 with NaOH). For experiments with mutants T623A, G648A, F656A and the corresponding WT control, the superfusate contained 94 mM KCl (with NaCl concentration correspondingly reduced) [25], [26]. Patch-pipettes (Corning 7052 glass, AM Systems, Carlsborg, USA) were pulled and heat-polished (Narishige MF83, Tokyo, Japan) to 2.5–4 MΩ; pipette dialysate contained (in mM): 130 KCl, 1 MgCl_2_, 5 EGTA, 5 MgATP, 10 HEPES (titrated to pH 7.2 using KOH) [14], [24], [25], [26]. Amiodarone (Sigma–Aldrich, Gillingham, UK) was dissolved in dimethyl sulfoxide to produce a stock solution of 50 mM, which was serially diluted to produce stock solutions ranging from 50 mM to 5 μM. The amiodarone stock solutions were then diluted 1:1000-fold with Tyrode solution to achieve concentrations stated in Section 3.

Measurements of hERG current (*I*_hERG_) were made at 37 ± 1 °C as described previously [14], [24], [25], [26], [27]. It has already been established that some of the mutant channels do not conduct adequate current using a traditional hERG protocol (depolarisation to +20 mV, followed by repolarisation to −40 mV), due to changes in the channel’s activation/inactivation kinetics, ion selectivity/sensitivity or expression level [20], [21], [28]. The selection of external [K^+^] and voltage-protocol for each mutant was informed by prior studies and experience. Activating voltage commands to +20 mV were used, with tail currents observed at either −40 mV (for most mutants), or −120 mV (T623A, V625A, G648A, F656A, V659A) [20], [21], [25], [26], [29], [30]. High external [K^+^] conditions were used for comparatively poorly expressing mutations (T623A, G648A, and F656A) [25], [26], [30]. For all mutants studied, block levels were attained by repetitive stimulation for 10 min and fractional inhibition of *I*_hERG_ tails measured. The data for each mutant were compared with WT *I*_hERG_ studied under comparable conditions; in all cases tail current measurements were evaluated (outward tail at −40 mV or inward tail at −120 mV with normal (4 mM) or raised (94 mM) [K^+^]) as in previous studies [25], [26], [27], [30].

Data were shown as mean ± SEM of the number of independent experiments indicated (*n*). Statistical comparisons were made using a Student *t* test or a one-way analysis of variance (ANOVA) followed by a Bonferroni post-test, as appropriate. *p* values <0.05 were considered statistically significant.

### 2.4 Concentration–response data and correction for *I*_hERG_ run-down

The fractional block (FB) of *I_hERG_* “tails” by the different drug concentrations studied was determined using the equation:

(1) $$\text{Fractional block}=1-((I_{\text{hERG-AMIOD}})/I_{\text{hERG-CONTROL}})$$

where “Fractional block” refers to the degree of inhibition of hERG current by a given concentration of amiodarone. *I*_hERG-AMIOD_ and *I*_hERG-CONTROL_ represent “tail” current amplitudes in the presence and absence of amiodarone.

Concentration–response data were fitted by a standard Hill equation of the form:

(2) $$\text{Fractional block}=1/(1+{\left({\text{IC}}_{50}/[\text{AMIOD}]\right)}^{h})$$

where IC_50_ is [AMIOD] producing half-maximal inhibition of the *I*_hERG_ tail and *h* is the Hill coefficient for the fit.

**Fig. 1:**
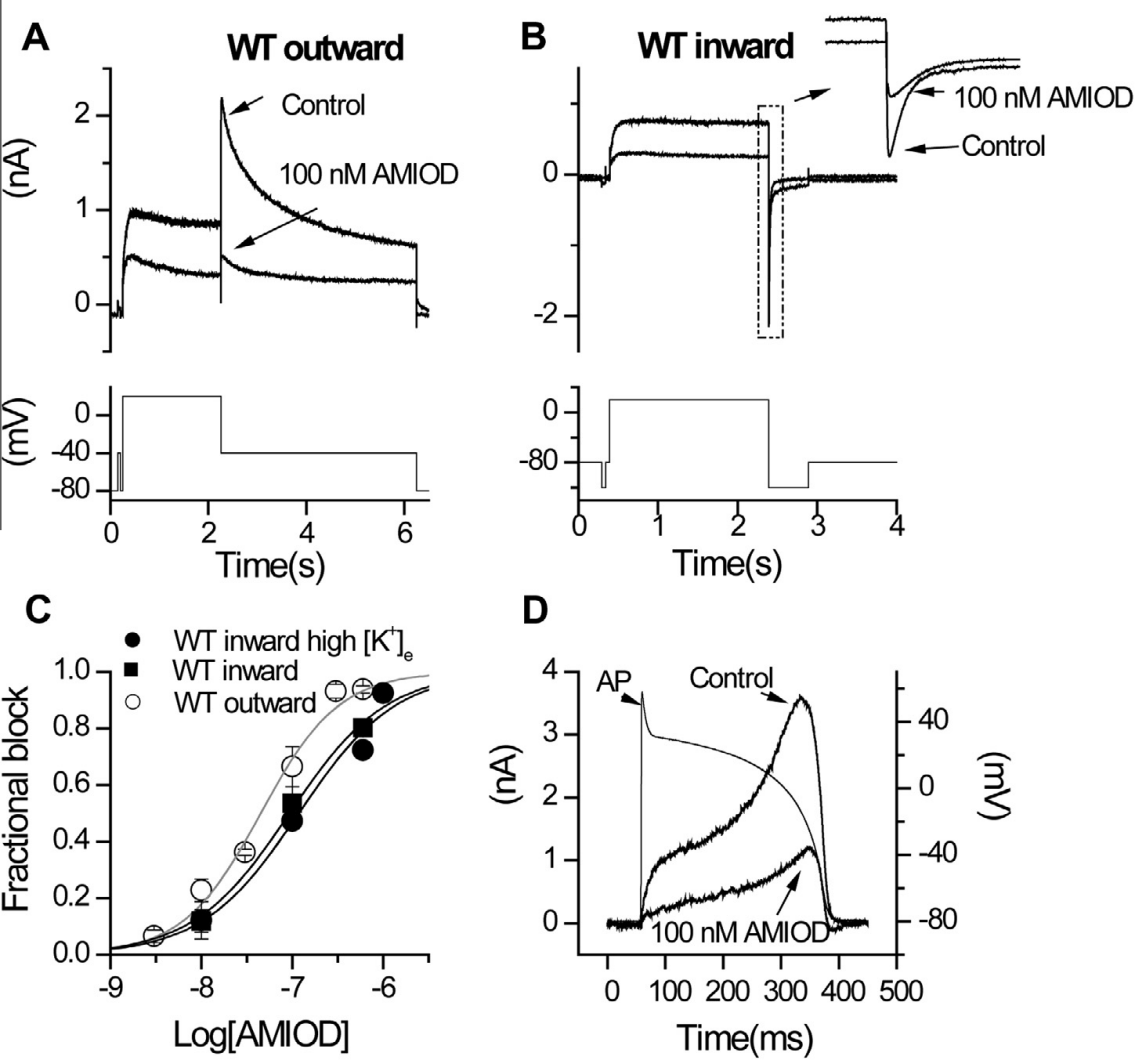
Effect of amiodarone on WT *I*_hERG_. (A, B) Representative current traces show outward (A) or inward WT *I*_hERG_ tail (B) in control (normal 4 mM [K^+^]_e_ Tyrode’s) solution and after 10 min application of 100 nM amiodarone (AMIOD), the current was evoked by the protocol shown in the lower panel and is shown on an expanded time-scale (denoted by the boxed area) in (B). Tail currents recorded at −40 mV or −120 mV were used to assess amiodarone inhibition. (C) Concentration response curves for outward and inward WT *I*_hERG_ tail inhibition by amiodarone in normal 4 mM [K^+^]_e_ and 94 mM [K^+^]_e_ Tyrode’s (high [K^+^]_e_)_._ Data were fitted with a Hill-equation (*n* ⩾ 5 cells per data-point). For IC_50_ and *h* values refer to Section 3, also see [14]. (D) Representative current traces in control (normal 4 mM [K^+^]_e_ Tyrode’s) solution and in 100nM amiodarone, overlying the applied AP voltage command.

As observed previously for amiodarone and its relatives [12], [14], amiodarone exhibited a progressive development of *I*_hERG_ blockade, reaching a stable level of block by ∼10 min of drug exposure, with continuous application throughout this period of the voltage protocol shown in Fig. 1A (start-to-start interval of 12 s). During this period, there was some overlying rundown of *I*_hERG_. Therefore, control experiments were performed to correct concentration–response data for *I*_hERG_ rundown. To do this, WT *I*_hERG_ was monitored during a 2–3 min stabilisation period followed by a 10-min recording period in normal Tyrode’s solution. The mean level of rundown of *I*_hERG_ tails following pulses to +20 mV during this 10 min period was 12.8% ± 1.8% of the peak outward tail magnitude (*n* = 5 cells). We subtracted 12.8% of current magnitude from the last tail current in the control periods and used the resulting value to calculate fractional block following (10 min) exposure to amiodarone. All concentration response data were run-down corrected, except for V659A, for which a clear pattern of run-down was absent. The correction procedure used for concentration response relations is in accord with that adopted previously for the study of amiodarone and its major metabolite desethylamiodarone [14].

### 2.5 Computational docking and molecular dynamics simulations

In the absence of a crystal structure for the hERG channel pore, computational docking of amiodarone to hERG was conducted using a homology model encompassing the pore helix, selectivity filter and S6 helix, built onto the crystal structure template of the MthK structure (pdb: 1LNQ) [31]. This model is described elsewhere [25], [32]. We recently showed that this model accords well with experimental data on drug block for a range of structurally-diverse hERG blockers [32]. Computational docking was conducted as described in [32] using the FlexiDock module of Sybyl (Certara, St. Louis, MO, USA) which allows unrestricted sampling of side chain bond rotations. Free side chain flexibility was sampled for the following residues: T623, S624, V625, Y652, F656 and S660. Definition of the drug-binding pocket, construction of starting configurations and choice of genetic algorithm parameters were carried out as described previously [25], [32]. A version of our hERG pore model including the S5 transmembrane helix (Dempsey et al., unpublished) was used for performing molecular dynamics simulations in a fully-hydrated bilayer membrane model to test the stability of amiodarone in its low energy score docked state and to explore amiodarone block of K^+^ diffusion and binding within the pore. Molecular dynamics simulations were done in a palmitoyl-oleoyl-phosphatidylcholine (POPC) bilayer membrane patch with 15 Å layers of water containing K^+^ and Na^+^ ions equivalent to a concentration of 140 mM above and below the membrane in a periodic boundary system with Gromacs [33] using methods described previously [34]. Structural figures and movies were made using Pymol [35] and VMD [36] respectively.

## 3 Results

### 3.1 *I*_hERG_ inhibition by amiodarone

The sensitivity of *I*_hERG_ to amiodarone was determined using the voltage protocol shown in Fig. 1A (continuously applied with a start-to-start interval of 12 s) [14], [25], [26]. Tail current magnitude at −40 mV was measured relative to instantaneous current observed during a brief (50 ms) depolarisation to −40 mV that preceded the +20 mV step depolarisation [14], [25], [26]. Fig. 1A shows representative traces in Control and in the presence of 100 nM amiodarone (AMIOD), which resulted in ∼70% inhibition of the *I*_hERG_ tail. The interaction of some drugs with hERG is influenced by the direction of K^+^ flux [12], [25], [26]. The effect of reversal of the direction of K^+^ ion flux on potency of amiodarone action was determined using the protocol shown in Fig. 1B (a 2 s depolarising step to +20 mV followed by a 500-ms hyperpolarising step to −120 mV), measuring inward *I*_hERG_ tails at −120 mV. As shown in the inset to Fig. 1B the extent of inward *I*_hERG_ tail inhibition by 100 nM amiodarone was less extensive than that seen for the outward tail current in Fig. 1A. A range of amiodarone concentrations was tested, for both outward and inward *I*_hERG_ tail inhibition, with concentration–response relations shown in Fig. 1C. The sensitivity to amiodarone of inward *I*_hERG_ in the presence of raised [K^+^]_e_ was also examined (this was necessitated by the requirement to have WT data under similar conditions as required to study some alanine mutants). The IC_50_ and *h* values derived from the fits to the data (Fig. 1C) were: outward tail 45.0 ± 5.2 nM, 1.0 ± 0.1; inward tail 93.3 ± 12.8 nM, 0.8 ± 0.1; inward tail with raised [K^+^]_e_ 117.8 ± 31.0 nM, 0.8 ± 0.2.

Sensitivity of WT *I*_hERG_ to amiodarone under ventricular action potential (AP) clamp was also determined (Fig. 1D; with the AP command applied at a start-to-start interval of 3 s). Maximal *I*_hERG_ during AP repolarisation was inhibited 65.5 ± 4.3% (*n* = 7) by 100 nM AMIOD, compared with 66.5 ± 7.0% (*n* = 5) with the standard protocol shown in Fig. 1A (*p* > 0.05, *t* test). The voltage at which peak *I*_hERG_ during repolarisation occurred was −20.6 ± 2.7 mV in control and −23.3 ± 2.4 mV in amiodarone (*p* > 0.05, *t* test).

### 3.2 The time-dependence of inhibition on *I*_hERG_ by amiodarone

A prior study, conducted utilising *Xenopus* oocyte expression, has suggested that hERG channel inhibition by amiodarone exhibits both gated-state and closed-state components [10]. However, we previously found that the closed-channel block component for *I*_hERG_ recorded from mammalian cells at physiological temperature was likely to be small for the amiodarone relative dronedarone [12]. We therefore investigated the issue of gated versus non-gated block for amiodarone using a similar approach to that previously adopted in studying dronedarone [12].

**Fig. 2:**
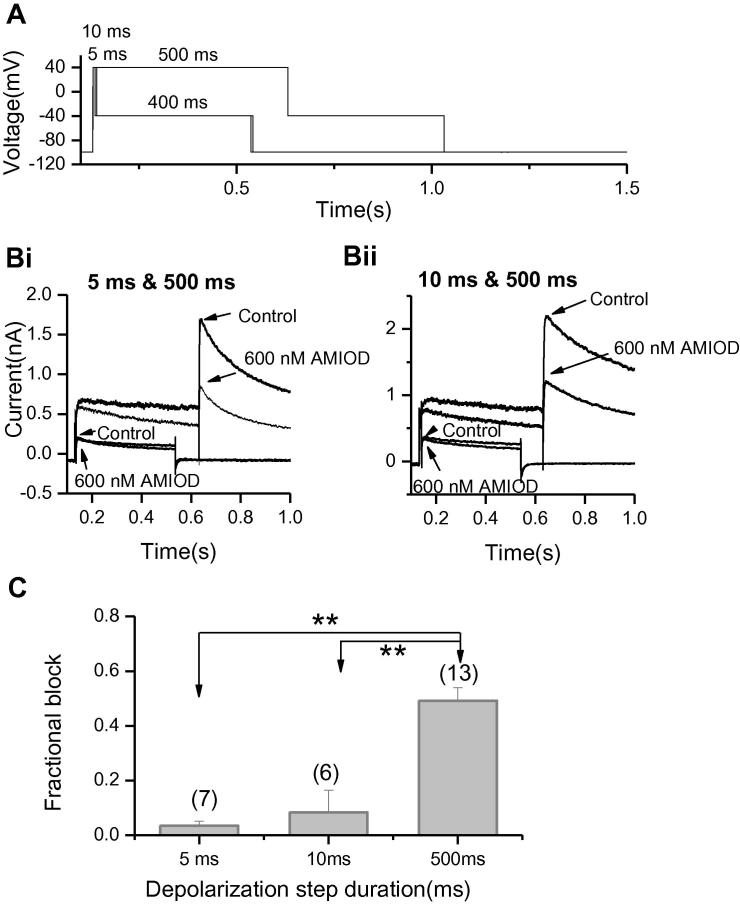
The time-dependence of inhibition of *I*_hERG_ by amiodarone. (A) Is a schematic representation of paired pulse voltage protocol used to elicit currents shown in (B) (Bi) shows representative current traces elicited during and following both 5 ms and 500 ms (*n* = 7) or (Bii) both 10 ms and 500 ms (*n* = 6, right) steps to +40 mV, in the absence and presence of 600 nM amiodarone (applied for 3 min in the absence of pulsing). (C) The bar chart displays the mean fractional block of *I*_hERG_ tails following the different duration steps to +40 mV. ^**^p < 0.001 compared to 500 ms step, one way ANOVA followed by Bonferroni’s post test (5 ms, *n* = 7; 10 ms, *n* = 6; 500 ms, *n* = 13).

During a sustained depolarisation (a 10 s step to 0 mV from a holding potential of −80 mV), *I*_hERG_ block showed progressive development with increased time during depolarisation, indicative of time-dependence of inhibition (data not shown), although this approach does not discriminate well between gated/non-gated inhibition over short time-periods. In order to investigate time-dependence of *I*_hERG_ inhibition over comparatively short time periods immediately following membrane depolarisation, the paired pulse protocol shown in Fig. 2A was used. This was applied from a holding potential of −100 mV, which greatly favours the closed channel state(s), and was comprised of two depolarising commands to +40 mV: the first of short duration (5 or 10 ms) and the second of longer duration (500 ms). The *I*_hERG_ tail at −40 mV after each command was measured. The protocol was applied under control conditions, was discontinued whilst the cells were exposed to 600 nM amiodarone for 3 min, and was then reapplied in the maintained presence of drug. As the channels were not gated through open/inactive states during the resting period during drug exposure, any block seen after the first brief (5 ms or 10 ms) depolarisation would be expected to result either from closed channel block or from very rapidly developing gated channel block. The channels were gated for longer during the 500 ms depolarisation. Representative traces are shown in Fig. 2B. We found the current traces in control and after amiodarone following 5 ms or 10 ms steps to +40 mV showed negligible difference (*p* > 0.05), but were substantially smaller in amiodarone following the 500 ms step. The bar chart in Fig. 2C displays the mean fractional block of *I*_hERG_ tails following 5 ms (*n* = 7), 10 ms (*n* = 6), and 500 ms (*n* = 13) steps to +40 mV. For 5 ms pulses tail current was inhibited by 3.4 ± 1.7% (*n* = 7); inhibition was 8.3 ± 8.1% for the 10 ms pulse (*p* > 0.05 compared with 5 ms pulse), with a marked increase to 48.6 ± 7.3% for the 500 ms pulse (*p* < 0.001 compared with both 5 ms and 10 ms). It is important to note that the protocol was applied only once in the presence of drug and so the mean values here do not represent steady-state block. However, the results from this experiment indicate clearly that *I*_hERG_ block by amiodarone is very largely gated-state dependent and that any component of closed channel block with the drug is likely to be small.

### 3.3 Alanine-scanning of potential amiodarone binding residues

**Fig. 3:**
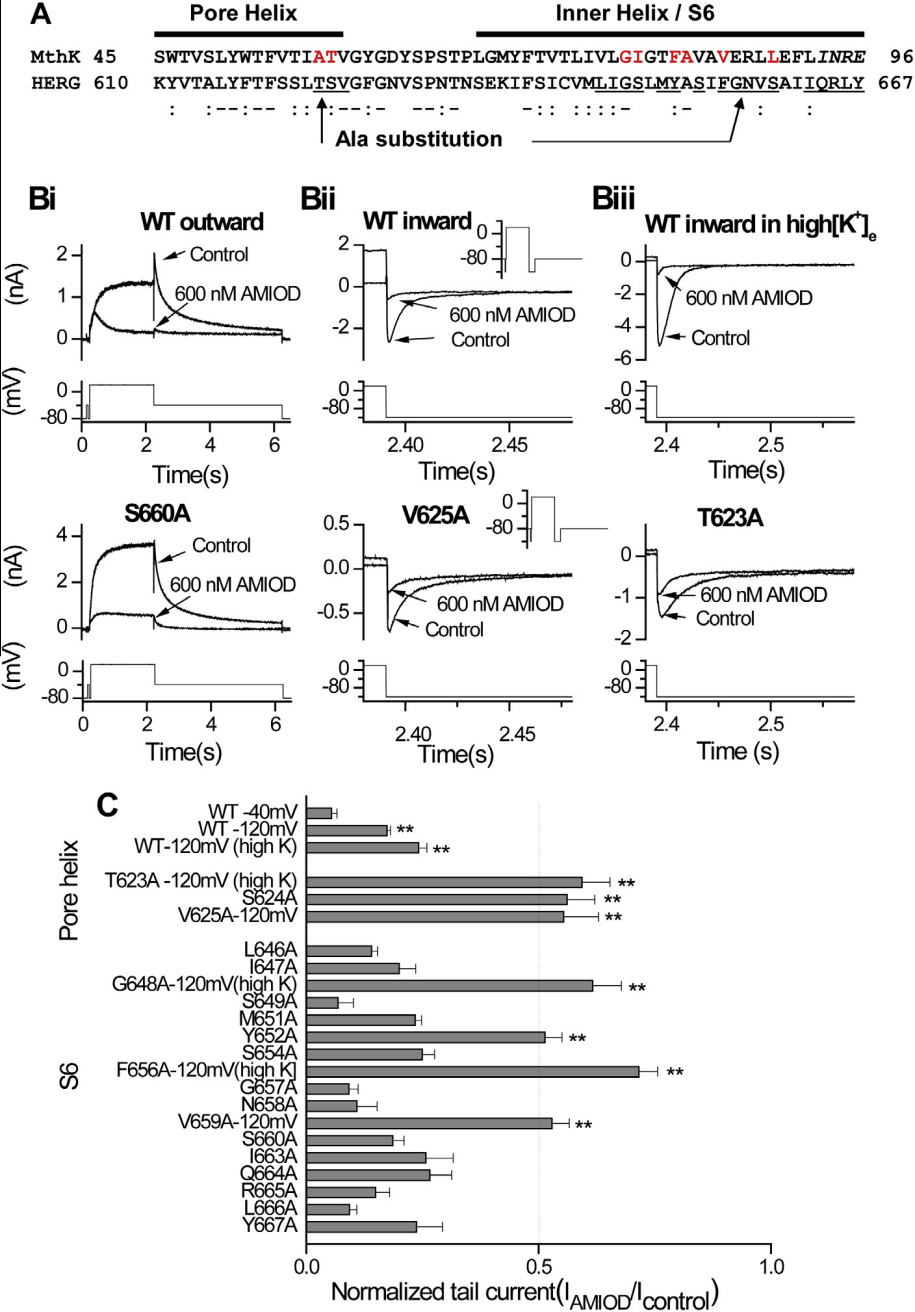
Alanine-scanning mutagenesis of hERG to define binding sites for amiodarone. (A) Sequence alignment for hERG and the MthK channel, highlighting the pore helix and S6 transmembrane domains. The residues of hERG analysed in this study by Ala-scanning mutagenesis are underlined. Bottom pair highlight amino acid identities (-), strong similarities (:). Amino acids in red text have side chains facing the pore cavity of the MthK structure. Note that the last four residues of S6 in the MthK structure italicised (*INRE*) are not seen in the crystal structure and aren’t included in the hERG model. (B) Example traces showing *I*_hERG_ inhibition of WT or mutants in transient transfected HEK293 cells, *I*_hERG_ was recorded before (control) and after achieving steady-state block of current with 600 nM amiodarone. Voltage protocols are shown in each lower panel. (C) Normalised current (*I*_AMIOD_/*I*_control_) measured after steady-state block by 600nM amiodarone (*n* = 5–6 for each point; error bars, ±SEM). A value of 1 indicates no current inhibition by amiodarone (^**^p < 0.001 compared to its WT, one way ANOVA followed by Bonferroni’s post test).

Key drug binding residues on the hERG channel reside in the S6 and pore helices of the channel [20], [21], [37]. We therefore conducted an alanine scan of pore helix and S6 residues (shown in Fig. 3A). Initial experiments utilised an amiodarone concentration at 600 nM; at steady state it produced 94.5 ± 0.0% block of WT outward tail current (Fig. 3Bi); 82.6 ± 0.0% block of inward tail current in normal [K^+^]_e_ and 75.8 ± 0.02% block of inward tail current in 94 mM high [K^+^]_e_ (Fig. 3Bi, Bii, and Biii, *p* < 0.001 compared with WT outward tail current). As shown in Fig. 3B (for S660A, V625A and T623A) inhibition of individual alanine mutants was compared to inhibition of WT *I*_hERG_ under similar recording conditions. The mean normalised remaining currents at steady state following drug application were calculated and plotted in Fig. 3C (*I*_AMIOD_/*I*_Control_), with larger values indicating smaller fractional block. As shown in Fig. 3C, *I*_hERG_ inhibition for each of the S6 domain mutants G648A, Y652A, F656A and V659A was statistically significantly different from the corresponding WT control. Three mutant channels located in the base of the pore helix (T623A, S624A, V625A) were also significantly less sensitive to amiodarone. To characterise further the relative importance of specific residues to the drug binding sites, we determined concentration–response relations for the 6 mutant channels least affected by drug.

### 3.4 Concentration-dependent *I*_hERG_ inhibition of the S6 domain mutations by amiodarone

**Fig. 4:**
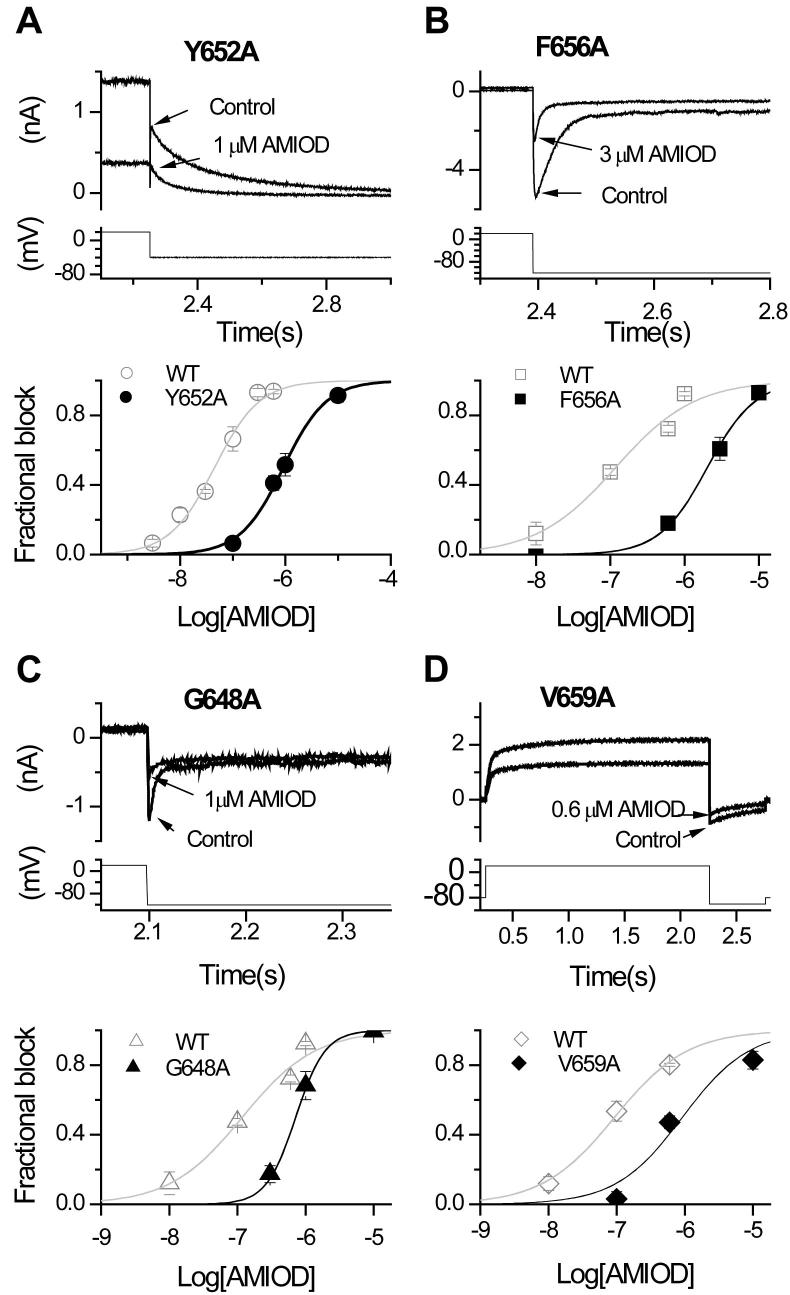
Effect of S6 mutations on amiodarone inhibition of *I*_hERG_. Representative traces from Y652A (A), F656A (B), G648A (C) and V659A (D) before (Control) and after achieving steady-state block by amiodarone, with the voltage protocol underneath. Lower panel shows concentration–response relation for the mutant (black) and its corresponding WT control (grey), yielding the IC_50_ and *h* values in section 3. (For all, *n* ⩾ 5 cells per data-point). Note that for some data-points in (A), (B), (C) the SEM values are small and obscured by the symbols.

Fig. 4A shows the effects of 1 μM amiodarone on Y652A hERG. This concentration, expected to produce well over 90% inhibition of WT *I*_hERG_ tails (see the concentration–response relation for outward *I*_hERG_ tails in Fig. 1C), produced ∼50% block of Y652A *I*_hERG_ (upper traces); the lower panel of Fig. 4A shows the mean concentration–response relations for Y652A *I*_hERG_ and for its WT control. The derived IC_50_ and *h* values for Y652A-hERG were 912.8 ± 61.3 nM and 1.1 ± 0.1, thus the IC_50_ was ∼20-fold its WT control. Fig. 4B (upper traces) shows representative traces for F656A *I*_hERG_ and its WT control; the lower panel shows corresponding concentration response relations. The derived IC_50_ and *h* values for F656A hERG were 2121.6 ± 168.6 nM and 1.4 ± 0.1: ∼17-fold its WT control. Fig. 4C and D show similar data for G648A hERG (IC_50_ and *h* of 673.9 ± 2.2 nM and 1.9 ± 0.0: ∼5.7-fold its WT control) and V659A hERG respectively (IC_50_ and *h* of 921.9 ± 498 nM, 0.9 ± 0.4: ∼9.9-fold its WT control).

### 3.5 Concentration-dependent *I*_hERG_ inhibition of the pore helix mutations by amiodarone

**Fig. 5:**
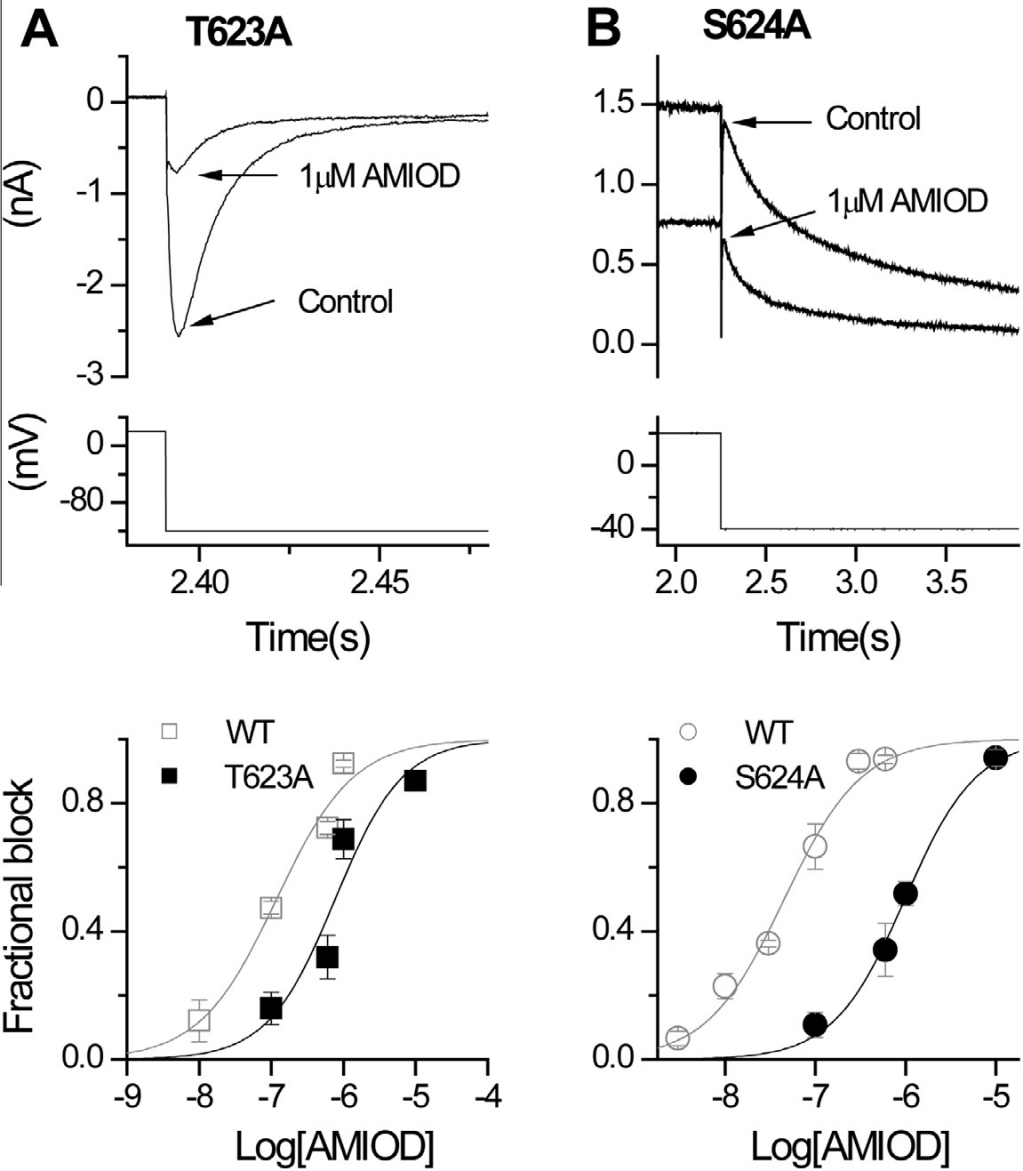
Effect of pore helix mutations on amiodarone inhibition of *I*_hERG_. Representative traces from T623A (A) and S624A (B) before (Control) and after achieving steady-state block by amiodarone, with the voltage protocol underneath. Lower panel shows concentration–response relation for the mutant (black) and its corresponding WT control (grey), yielding the IC_50_ and *h* values in Section 3. (For all, *n* ⩾ 5 cells per data-point.) Note that for some data-points the SEM values are small and obscured by the symbols.

T623A and S624A hERG were also studied but V625A was not included in full concentration–response studies. This is because, although the alanine scan identified V625 to influence amiodarone block, under our conditions it was found to be technically difficult to maintain sufficiently sustained recordings from V625A *I*_hERG_ to obtain full concentration–response data for amiodarone. Fig. 5A shows data for T623A hERG. 1 μM amiodarone blocked inward *I*_hERG_ by 68.8 ± 6.1%, with concentration response data yielding IC_50_ and *h* values of 765.5 ± 287.8 nM and 0.9 ± 0.4. S624A hERG can be studied under similar conditions to WT at normal [K^+^]_e_ and Fig. 5B shows representative traces for the effect of 1 μM amiodarone and the corresponding concentration–response relation, yielding IC_50_ and *h* values of 979.2 ± 84.3 nM and 1.1 ± 0.1. The IC_50_ for T623A hERG was ∼6.5-fold its WT control and for S624A hERG was ∼21.7-fold its WT control. Table 2 summarises experimental data from all the mutants for which full concentration–response relations were obtained.

### 3.6 Docking of amiodarone into a hERG pore homology model

**Fig. 6:**
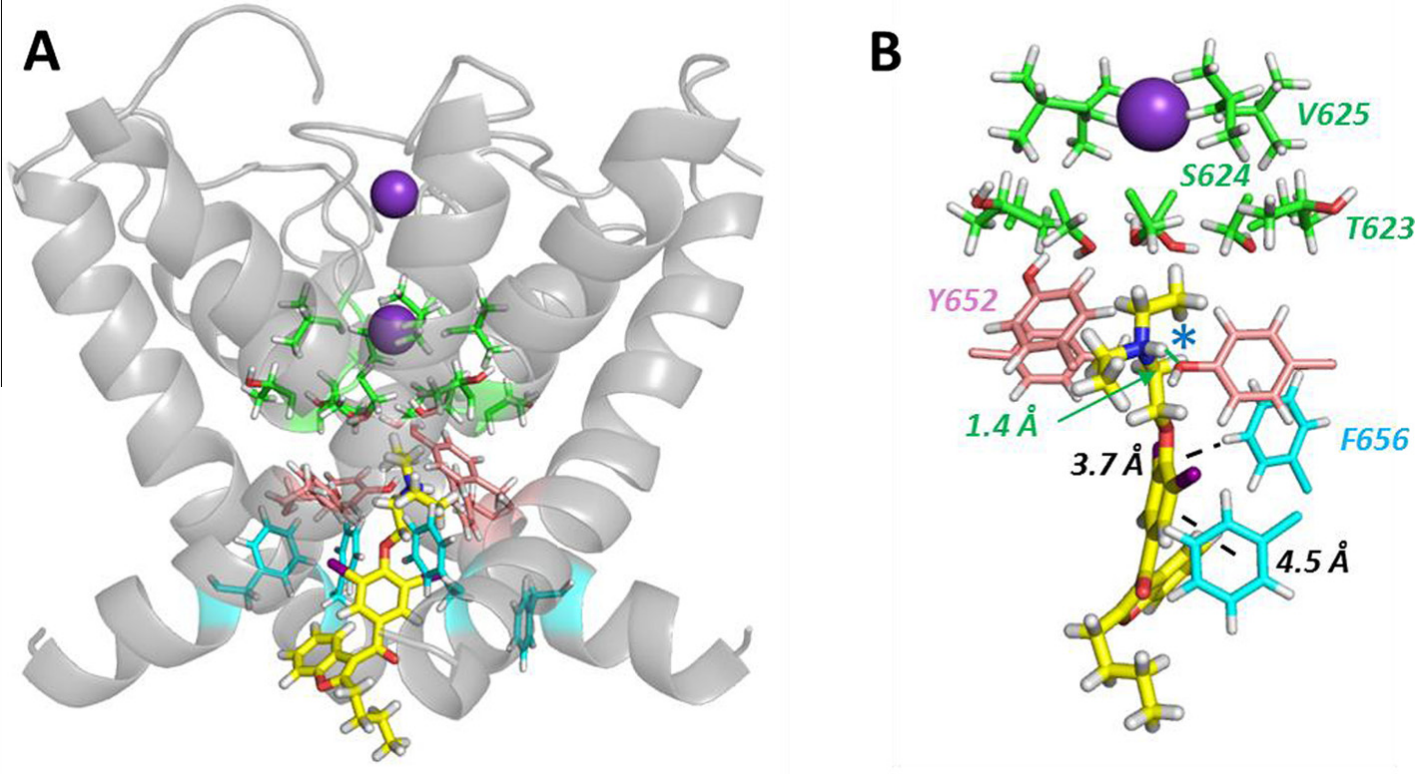
Representative low energy score docking output for amiodarone in the MthK-based hERG pore homology model. (A) Amiodarone is shown in relation to the amino acid residues described in the text: blue: F656, pink: Y652; green: T623, S624, V625. These residues are also annotated in (B) which highlights the set of interactions between amiodarone and specific amino acid side chains including two pi-stacking interactions between F656 and amiodarone aromatic rings, and two cation–pi interactions and one hydrogen bond involving the protonated amino group and Y656 side chains. The location of the aliphatic amino group near the internal binding site for a K^+^ ion is indicated by the blue star. Stabilisation of the protonated amino group in this location may be enhanced by the hydroxyl side chain groups of S624. (For interpretation of the references to colour in this figure legend, the reader is referred to the web version of this article.)

Docking of amiodarone into the MthK-based homology model of the hERG pore resulted in drug-bound states that are broadly consistent with the experimental data (Fig. 6). The predominant conformational state from docking using FlexiDock was one in which the drug was oriented with the tertiary aliphatic amino group near the top of the channel pore cavity, in or near the internal binding site for a K^+^ ion [31], [38] and the bulky iodinated aromatic group lower down in the cavity; a representative structure is shown in Fig. 6. In this state the drug makes multiple interactions with the aromatic side chains of Y652 and F656, consistent with the reduction in drug block in hERG Y652A and F656A (Fig. 3, Fig. 4). The location of the protonated tertiary aliphatic amino group near the internal K^+^ binding site is consistent with the effect of inward K^+^ flux in reducing amiodarone block potency (Fig. 1C) as a result of direct competition of drug and K^+^ for binding in the pore cavity. The location of amiodarone high in the pore cavity with the protonated tertiary amino group located just below the selectivity filter near S624 is also consistent with the reduction in amiodarone block in hERG S624A (Figs. 3C and 5). These interpretations are supported by molecular dynamics simulations of amiodarone in the low energy score docked conformation within a membrane-embedded MthK model extended to include the S5 helix (Movies 1 and 2). In the absence of drug, K^+^ ions were observed to diffuse into the pore cavity through the open gate on the cytoplasmic side of the channel and periodically to occupy the internal K^+^ binding site (Movie 1). The bound configuration of amiodarone within the channel pore was found to be stable (Movie 2) and in this location the drug blocked K^+^ ions from interacting with the internal K^+^ binding site, and indeed entirely blocked K^+^ ions from entering the pore cavity.

## 4 Discussion

### 4.1 Clinical relevance

Previous experiments using *Xenopus* oocytes yielded an amiodarone IC_50_ value for *I*_hERG_ of 9.8 μM [10] whilst in mammalian expression systems *I*_hERG_ IC_50_ values between ∼26 and 300 nM were reported [12], [13], [14], [16], [17]. Amiodarone is highly lipophilic and for such agents the use of *Xenopus* oocytes can markedly underestimate blocking potency due to drug accumulation in the yolk sac [39], [40]. Amiodarone has also been shown to produce greater *I*_hERG_ block at physiological (37 °C) than at ambient (23 °C) temperature (IC_50_ of 0.30 μM versus 0.56 μM, respectively) [17]. Our IC_50_ of ∼45 nM is consistent with the potency of inhibition observed previously [12], [13], [14], [16], [17]. The comparable levels of WT *I*_hERG_ inhibition observed here with conventional and AP voltage clamp (Fig. 1) is predictive of significant inhibition of *I*_Kr_ during physiological waveforms within the plasma clinical concentration range (1.6–5.9 μM) [41]. Prior data from *Xenopus* oocyte experiments suggest that, at a holding potential of −80 mV, recovery of *I*_hERG_ from block between successive commands in the presence of drug would be anticipated to be small at cycle lengths of ∼10 s or less [42]. Our data are consistent with this, as the AP and step protocols used in Fig. 1 achieved similar levels of block despite differences not only in waveform type, but also in protocol application frequency. Thus, little recovery of *I*_hERG_ from block would be anticipated at physiological heart rates. Recently, results have been reported that some ion channel effects of amiodarone that underlie the drug’s clinical actions may result from physical effects of the drug on the lipid bilayer in which ion channels reside [43]. The structure–functional analysis in the present study indicates that *I*_hERG_ channel inhibition (and consequently the associated Class III effect of the drug) results from a direct channel-drug interaction within the channel pore and not from a physical effect of the drug on the lipid bilayer.

### 4.2 Mechanism of WT *I*_hERG_ block

Amiodarone has been reported to block hERG channels expressed in *Xenopus* oocytes in closed, open, and inactivated states [10]. Whilst the electrophysiological discrimination between closed and rapid open state channel block can be challenging [27], [44], the use of protocols similar to that shown in Fig. 2A can provide some clarification in discriminating gated (open/inactivated) from closed state inhibition [12]. Thus, we observed negligible block of *I*_hERG_ tails when these were elicited by 5 or 10 ms brief commands in the presence of amiodarone. Additionally, with the protocol employed in Fig. 2, currents in the absence and presence of amiodarone elicited by 500 ms commands initially overlaid one another and then diverged as *I*_hERG_ block developed during the depolarisation (Fig. 2Bi, Bii). It is likely, therefore, that any closed channel block component, if present, is slight. Prior studies have demonstrated that amiodarone inhibition of *I*_hERG_ shows a moderate dependence on inactivation that is intermediate between that of Class I antiarrhythmic drugs (low) and other Class III methansulphonanilides (high) [13], [22]. Titration of the level of *I*_hERG_ inactivation through the use of single and double mutations that impair inactivation resulted in graded changes to amiodarone IC_50_: the N588 K and S631A mutations each resulted in IC_50_ values 4-fold that for WT *I*_hERG_, whilst the N588 K/S631A double mutation resulted in an IC_50_ value 29-fold that of WT *I*_hERG_ [13]. Thus, when the data from the present study are considered alongside results of prior studies [10], [13], [22], gated state block is likely to involve interactions with both activated and inactivated channels. Although *I*_hERG_ inactivation is reduced in high [K^+^]_e_ [45], the reduced sensitivity (increased IC_50_) for *I*_hERG_ block by amiodarone for inward *I*_hERG_ tail current with both normal as well as raised [K^+^]_e_ suggests that the effect of reversing the direction of K^+^ flux on blocking potency is likely to result from a direct interaction between K^+^ ions and amiodarone, rather than a consequence of altered inactivation [25]. Moreover, a direct interaction involving electrostatic repulsion or “knock-off” [45], [46] is consistent with amiodarone binding within the K^+^ ion conduction pathway, supported by the results of our docking analysis (Fig. 6) and MD simulations (Movies 1 and 2).

### 4.3 Molecular determinants of block

The apparently large size of the central cavity below the selectivity filter, and the positioning of the aromatic side chains of Y652 and F656 on S6 allow hERG to accommodate diverse drugs [18], [20]. Although we have previously identified a partial dependence of amiodarone block on aromatic residues within the hERG channel cavity [12], to our knowledge, the present study is the first to make an extensive and quantitative description of the side chains in the hERG channel cavity that constitute determinants of amiodarone block. This study locates the binding site of the drug within the K^+^ permeation pathway below the selectivity filter. Mutation to alanine of T623, S624, V625 located near or within the selectivity filter, and G648, Y652, F656, V659 in the S6 helix, all attenuated amiodarone block (Fig. 3C). These mutations are similar to those attenuating block by the methanesulphonanilides E-4031, MK-499, dofetilide and ibutilide [20], [21], [23], [47], but differ somewhat from those for terfenadine and cisapride, for which high affinity block was little affected in V625A and G648A mutants [48]. Although the binding residues for gated-state hERG inhibitors generally involve combinations of those investigated here, it has been noted previously that the relative importance of particular residues can vary between compounds [21], [48]. The results of the present study agree with this notion, though direct quantitative comparisons with previous analyses of the molecular determinants of *I*_hERG_ block by other drugs is limited by the fact that alanine scanning of the hERG pore cavity has often utilised only a single (profound-blocking) concentration [12], [21], [23], [48] and/or not all the residues studied here have been investigated [49]. Full IC_50_ determination for drug block of a range of hERG alanine mutants has been described for MK-499, terfenadine, cisapride [20], clofilium and ibutilide [50] in *Xenopus* oocytes at room temperature.

Perhaps the most significant feature of amiodarone binding compared to other high affinity blockers (see Table 3) is the smaller effect of alanine replacement of either of Y652 and F656 (17–20-fold increases in IC_50_ for block) than has been seen previously for high affinity methanesulphonanilides. For example, Lees-Miller et al. reported the IC_50_ for dofetilide block of hERG F656V to be ∼120-fold that of WT [47], whilst Mitcheson et al. reported values of 650-fold and 94-fold WT respectively, for F656A and Y652A mutations [20]. Subsequent work identified substantial attenuation of *I*_hERG_ block by both dofetilide and E-4031 with Y652A and F656A mutations at single (high) drug concentrations [21]. IC_50_ values for *I*_hERG_ block by cisapride and terfenadine were also substantially elevated by Y652A and F656A mutations [20], [51]. A striking feature of our results is the similar effect of pore-helix/selectivity filter mutations and S6 aromatic mutations on amiodarone inhibition of *I*_hERG_. Thus, the relative importance for amiodarone binding (based on measured IC_50_ values, so excluding V625A) of the residues studied here is: S624A ∼ Y652A > F656A > V659A > G648A > T623A. This compares with F656≫Y652 > G648 = V625 > T623 > S624 = V659 for MK-499 [20].

### 4.4 Computational docking and molecular dynamics simulations

**Fig. 7:**
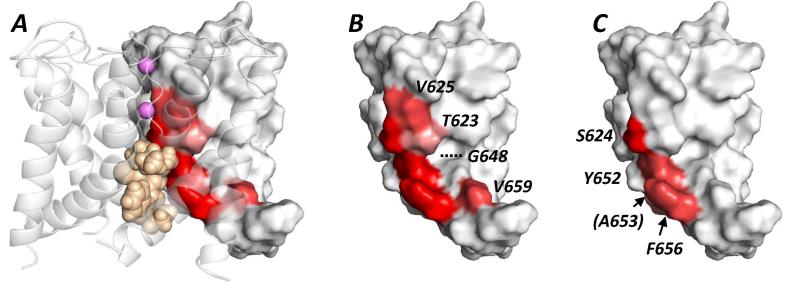
Comparison of experimental and computational analysis of amiodarone block. (A) Low energy score structure of amiodarone (wheat coloured space filling representation) docked into the MthK-based hERG model extended to include the S5 helix. One subunit of the model is rendered as a Connelly surface coloured as a heat map according to amino acids whose mutation to Ala attenuates drug binding as defined in panel (B). K^+^ ions in the S1 and S3 positions of the selectivity filter are pink spheres. (B) One subunit of the hERG model extracted from panel (A) and coloured to define residues whose Ala mutation attenuates amiodarone block by: 17–22-fold (deep red); approx. 10-fold (pale red); 5–7-fold (mauve); the latter group comprised T623 and G648 (Table 2), however G648 lies behind Y652 and is hidden in this view. (C) The same subunit coloured according to residues that make interactions (as defined in [32]) with amiodarone in low energy docked states. Annotations in panel C define residues that make direct interactions with drug in docking and whose mutation to Ala attenuates drug block (except for A653 which was not mutated experimentally). Annotations in panel (B) define residues whose Ala mutation attenuates amiodarone block but which do not make direct interaction with drug in low energy score docked states. (For interpretation of the references to colour in this figure legend, the reader is referred to the web version of this article.)

Comparison of residues that make defined interactions with amiodarone in docked states (Fig. 6) with those having reduced amiodarone block in the alanine scan (Fig. 3; Table 2), identifies side chains for which the effects of alanine replacement are likely to result from direct interaction with the drug. The patch of molecular surface defined by residues affecting amiodarone sensitivity in the alanine scan (Fig. 7B) is considerably larger than the molecular surface of amiodarone (Fig. 7A) and these residues cannot all simultaneously interact with drug. Direct interaction between amiodarone and S624, Y652 and F656 likely accounts for the marked attenuation of amiodarone block in S624A, Y652A and F656A, respectively. The effects of V625A, G648A and V659A are likely to result from indirect (allosteric) effects on amiodarone binding. The reduction in sensitivity of V625A to amiodarone may result from conformational perturbation around the selectivity filter, which disrupts ion selectivity and inactivation, and/or repositioning of S624 so that it is not optimally oriented for high-affinity binding [50], [52]. Whilst the V659A mutation reduces the potency of many compounds [53], this side chain is likely to be oriented away from the pore cavity, assuming that the S6 helix retains a helical conformation through this sequence (Fig. 7B). This indicates that this residue is unlikely to be a direct binding determinant. Indeed, the V659A mutation significantly alters gating, shifting the voltage dependence of activation by −30 mV and reduces drug block potency by increasing rates of recovery from block between pulses [53]. These effects may be mediated via interactions with the S5 helix. The reduction in amiodarone block of G648A hERG is similar to the effect of this mutation on high affinity blockers such as dofetilide, ibutilide and MK-499 but not cisapride [53]. Larger residues in this position may alter the positioning of other inner cavity residues [53], [54], so the reduction in amiodarone sensitivity may be due to an allosteric effect of this mutation. The potential for indirect (allosteric) effects of mutations on drug binding highlights the value of considering the results of mutagenesis experiments alongside structural and docking information, as provided by Fig. 6, Fig. 7 and molecular dynamics Movies 1 and 2 in this study.

The low energy score conformation of amiodarone with the aliphatic amino group near the cavity K^+^ binding site just below S624 (Fig. 6) is stable during MD simulations within the membrane-embedded pore model (Movie 2). This orientation of amiodarone is consistent with the effects on drug block of a series of amiodarone analogues with modified substituents around the amino group that produced marked changes in IC_50_ values for hERG block [55]. A positively charged amino group is required for high blocking efficacy [55] and this likely reflects a location of this group beneath the selectivity filter where interactions with S624 and Y652 side chains, and the cavity K^+^ binding site are possible. Thus, high affinity block of *I*_hERG_ by amiodarone is favoured by drug binding high in the pore cavity within the K^+^ ion conduction pathway and interacting with S624 as strongly as with Y652.

## Conflicts of interest

None.

## Figures and Tables

**Table 1:** Mutagenic primers for alanine mutants in S6 helix of hERG.

Mutant	Primer sequence (5′–3′)
L646A	Forward: CATCTGCGTCATGGCCATTGGCTCCCTC
Reverse: GAGGGAGCCAATGGCCATGACGCAGATG

I647A	Forward: CATCTGCGTCATGCTCGCTGGCTCCCTCATGTATG
Reverse: CATACATGAGGGAGCCAGCGAGCATGACGCAGATG

G648A	Forward: CGTCATGCTCATTGCCTCCCTCATGTATG
Reverse: CATACATGAGGGAGGCAATGAGCATGACG

S649A	Forward: GTCATGCTCATTGGCGCCCTCATGTATGC
Reverse: GCATACATGAGGGCGCCAATGAGCATGAC

M651A	Forward: GCTCATTGGCTCCCTCGCGTATGCTAGCATCTTCG
Reverse: CGAAGATGCTAGCATACGCGAGGGAGCCAATGAGC

S654A	Forward: CTCATGTATGCTGCCATCTTCGG
Reverse: CCGAAGATGGCAGCATACATGAG

G657A	Forward: GCTAGCATCTTCGCCAACGTGTCGG
Reverse: CCGACACGTTGGCGAAGATGCTAGC

N658A	Forward: GCTAGCATCTTCGGCGCAGTGTCGGCCATCATC
Reverse: GATGATGGCCGACACTGCGCCGAAGATGCTAGC

V659A	Forward: CATCTTCGGCAACGCGTCGGCCATCATCC
Reverse: GGATGATGGCCGACGCGTTGCCGAAGATG

S660A	Forward: CTTCGGCAACGTGGCGGCCATCATCC
Reverse: GGATGATGGCCGCCACGTTGCCGAAG

I663A:	Forward: GTCGGCCATCGCCCAGCGGCTG
Reverse: CAGCCGCTGGGCGATGGCCGAC

Q664A	Forward: GTCGGCCATCATCGCGCGGCTGTACTCG
Reverse: CGAGTACAGCCGCGCGATGATGGCCGAC

R665A	Forward: CCATCATCCAGGCGCTGTACTCGG
Reverse: CCGAGTACAGCGCCTGGATGATGG

L666A	Forward: CCATCATCCAGCGGGCGTACTCGGGCACAG
Reverse: CTGTGCCCGAGTACGCCCGCTGGATGATGG

Y667A	Forward: CATCCAGCGGCTGGCCTCGGGCACAGCC
Reverse: GGCTGTGCCCGAGGCCAGCCGCTGGATG

**Table 2:** Effect of pore helix and S6 mutations on *I*_hERG_ inhibition by amiodarone.

Channel	Voltage step (mV)	K^+^ (mM)	Tested amiod concentration range (*n* numbers per concentration) (nM)	IC_50_ (mean ± SEM) (nM)	*h*	Shift in potency compared to its WT-control	Shift in WT potency compared to WT-1
WT-1	−40	4	3–600 (5–6)	45.0 ± 5.2	1.0 ± 0.1		
WT-2	−120	4	10–600 (5)	93.3 ± 12.8	0.8 ± 0.1		2.1
WT-3	−120	94	10–1000 (5)	117.8 ± 31.0	0.8 ± 0.2		2.6
T623A	−120	94	100–10,000 (5–6)	765.5 ± 287.8	0.9 ± 0.4	6.5	
S624A	−40	4	100–10,000 (5–6)	979.2 ± 84.3	1.1 ± 0.1	21.8	
G648A	−120	94	100–10,000 (4–6)	673.9 ± 2.2	1.9 ± 0.0	5.7	
Y652A	−40	4	100–10,000 (5–6)	912.8 ± 61.3	1.1 ± 0.1	20.3	
F656A	−120	94	10–10,000 (5–8)	2021.6 ± 168.6	1.4 ± 0.1	17.2	
V659A	−120	4	100–10,000 (5)	921.9 ± 498.0	0.9 ± 0.4	9.9	

**Table 3:** Comparison of IC_50_ fold change to WT in pore helix and S6 mutants for some high affinity hERG inhibitors.

Agents	Cell line	Recording temperature	WT IC_50_ (nM)	IC_50_ fold increase (mutant IC_50_/WT IC_50_)							Ref.
Pore helix			S6			
T623A	S624A	V625A	G648A	Y652A	F656A	V659A
Amiodarone	HEK293	37 °C	45	6.5	22	6 (E)	5.7	20	17	9.9	This paper
Clomipramine	HEK293	36 °C	130								[56]
Oocyte	Room temperature	12,400					6	12		
Cisapride	Oocyte	Room temperature	133			2	1	100	40		[20]
Clofilium	Oocyte	Room temperature	30	12	381	250		1329	484		[23], [50]
Dofetilide estimated	Oocyte	Room temperature	420	7	9	130	171	25	62	3	[21]
E-4031 estimated	Oocyte	Room temperature	570	4	13	86	40	31	89	4	[21]
Ibutilide	Oocyte	Room temperature	28	54	93	>300	140 (E)	67 (E)	140 (E)	18 (E)	[23]
MK-499	Oocyte	Room temperature	34	5		54	55	94	650		[20]
Terfenadine	Oocyte	Room temperature	134			1.5	1.5	150	100		[20]
Verapamil	HEK293	Room temperature	143								[57], [58]
Oocyte		5100					16	20		
Ziprasidone	HEK293	37 °C	120								[59]
Oocyte	Room temperature	2800					140	357		

E: estimated. This table shows the effects of mutations in the pore-helix/selectivity filter region (T623A, S624A, V652A) and S6 helix (G648, Y652A, F656A, V659A) on *I*_hERG_ block by some high affinity hERG inhibitors, the fold change in IC_50_ relative to its corresponding WT control is given as IC_50_ mutant/IC_50_ WT. Where “estimated” fold change values are given, experimentally derived IC_50_ values were not given and so the estimated values used here were calculated using single dose data available in the relevant paper, by using a standard Hill equation (Eq. (2)): fractional block = 1/(1 + (IC_50_/[drug])*^h^*), and assuming *h* = 1.

## Appendix A Supplementary data

Supplementary Movie 1 Starting structure and molecular dynamics simulation of the drug-free MthK-based hERG pore model. The first 3 s illustrates the simulation starting structure with the pore model embedded in a lipid bilayer and the location of K^+^ (pink) and Cl^−^ ions (yellow) equivalent to a concentration of 140 mM KCl; waters have been removed for clarity. The rest of the simulation illustrates the time evolution of the pore model and K^+^ ions (the lipids and Cl^−^ ions are hidden) during 200 ns of molecular dynamics. Selected K^+^ ions are given unique colours to illustrate the diffusional exchange of ions into the cavity K^+^ binding site during the simulation.

Supplementary Movie 2 Equivalent simulation system as Movie 1 but containing a molecule of amiodarone docked into the pore cavity in its low energy score configuration in the starting structure (see Figs. 6 and 7 of main text). During the simulation amiodarone retained a stable configuration within the pore cavity and blocked diffusion of K^+^ ions into the pore and the cavity K^+^ binding site.
