# Supplementary figures and images for: Interactions between amiodarone and the hERG potassium channel pore determined with mutagenesis and in silico docking
Source: Biochem Pharmacol. 2016 Aug 1;113:24–35. doi: 10.1016/j.bcp.2016.05.013 (PMC4959829; doi:10.1016/j.bcp.2016.05.013)

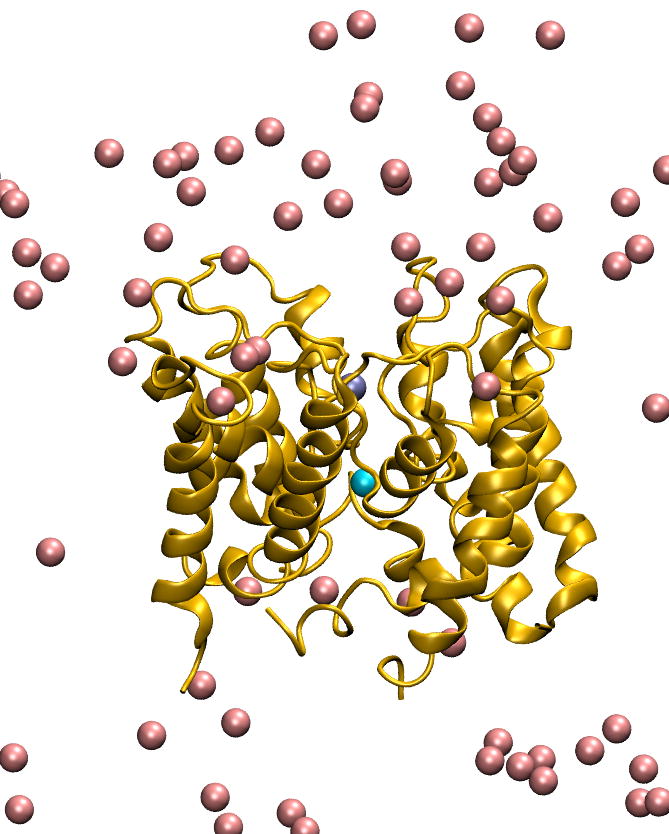

Supplement: Supplementary Movie 1 — Starting structure and molecular dynamics simulation of the drug-free MthK-based hERG pore model. The first 3 s illustrates the simulation starting structure with the pore model embedded in a lipid bilayer and the location of K+ (pink) and Cl− ions (yellow) equivalent to a concentration of 140 mM KCl; waters have been removed for clarity. The rest of the simulation illustrates the time evolution of the pore model and K+ ions (the lipids and Cl− ions are hidden) during 200 ns of molecular dynamics. Selected K+ ions are given unique colours to illustrate the diffusional exchange of ions into the cavity K+ binding site during the simulation. [file mmc1.jpg]

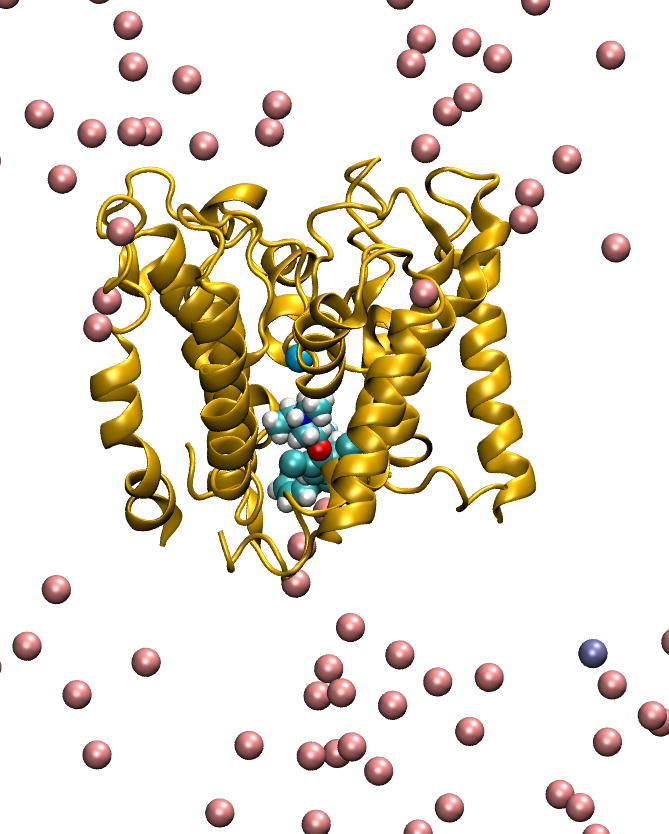

Supplement: Supplementary Movie 2 — Equivalent simulation system as Movie 1 but containing a molecule of amiodarone docked into the pore cavity in its low energy score configuration in the starting structure (see Figs. 6 and 7 of main text). During the simulation amiodarone retained a stable configuration within the pore cavity and blocked diffusion of K+ ions into the pore and the cavity K+ binding site. [file mmc2.jpg]
